# Comparison of outcomes in hematological malignancies treated with haploidentical or HLA-identical sibling hematopoietic stem cell transplantation following myeloablative conditioning: A meta-analysis

**DOI:** 10.1371/journal.pone.0191955

**Published:** 2018-01-30

**Authors:** Dangui Chen, Di Zhou, Dan Guo, Peipei Xu, Bing Chen

**Affiliations:** 1 Department of Hematology, Nanjing Drum Tower Hospital Clinical College of Nanjing Medical University, Nanjing, People’s Republic of China; 2 Department of Hematology, Nanjing Drum Tower Hospital, The Affiliated Hospital of Nanjing University Medical School, Gulou district, Nanjing, People’s Republic of China; University of Kentucky, UNITED STATES

## Abstract

**Purpose:**

Haploidentical and human leukocyte antigen (HLA)-identical sibling hematopoietic stem transplantation are two main ways used in allogeneic hematopoietic stem cell transplantation (allo-HSCT). In recent years, remarkable progress has been made in haploidentical allo-HSCT (HID-SCT), and some institutions found HID-SCT had similar outcomes as HLA-identical sibling allo-HSCT (ISD-SCT). To clarify if HID-SCT has equal effects to ISD-SCT in hematologic malignancies, we performed this meta-analysis.

**Methods:**

Relevant articles published prior to February 2017 were searched on PubMed. Two reviewers assessed the quality of the included studies and extracted data independently. Odds ratio (OR) and 95% confidence intervals (CIs) were calculated for statistical analysis.

**Results:**

Seven studies including 1919 patients were included. The rate of platelet engraftment is significantly lower after HID-SCT versus ISD-SCT while there is no difference in neutrophil engraftment (OR = 2.58, 95% CI = 1.70–3.93, *P <* 0.00001). The risk of acute graft-versus-host disease (GVHD) is significantly higher after HID-SCT versus ISD-SCT (OR = 1.88, 95% CI = 1.42–2.49, *P <* 0.00001), but the relapse rate is lower in HID-SCT group (OR = 0.70, 95% CI = 0.55–0.90, *P* = 0.005). The incidence rates of overall survival (OS) and disease-free-survival/leukemia-free survival/relapse-free survival (DFS/LFS/RFS) after ISD-SCT are all significantly superior to HID-SCT (OR = 1.32, 95% CI = 1.08–1.62, *P* = 0.006; OR = 1.25, 95% CI = 1.03–1.52, *P* = 0.02). There is no significant difference in transplantation related mortality (TRM) rate after HID-SCT and ISD-SCT.

**Conclusion:**

After myeloablative conditioning, patients receiving ISD-SCT have a faster engraftment, lower acute GVHD and longer life expectancy compared to HID-SCT with GVHD prophylaxis (cyclosporine A, methotrexate, mycophenolate mofetil and antithymoglobulin; CsA + MTX + MMF + ATG). Currently, HID-SCT with GVHD prophylaxis (CsA + MTX + MMF + ATG) may not replace ISD-SCT when HLA-identical sibling donor available.

## Introduction

Allogeneic hematopoietic stem cell transplantation (allo-HSCT) with human leukocyte antigen (HLA)-identical sibling or unrelated donor is the main way for treatment for high-risk hematological malignancies. For patients without a suitable donor, especially those in urgent need of transplantation, haploidentical allo-HSCT (HID-SCT) is an option [1]. HID-SCT was unsuccessful for many years because of graft rejection and high incidence of acute graft-versus-host disease (GVHD), but the progress in GVHD prophylaxis and conditioning regimen has made HID-SCT possible [2,3]. A multicenter phase-2 study from the Chinese Bone Marrow Transplant Cooperative Group (CBMTCG) showed that the combination of cyclosporine A, methotrexate, mycophenolate mofetil (CsA + MTX + MMF) for GVHD prophylaxis significantly decreased the incidence of acute GVHD without an increase in relapse or any adverse impact on survival in standard-risk patients compared with historical controls in ISD-SCT [4]. Similarly, ATG deletes T lymphocytes chronically in vivo, and prevents GVHD without increasing the risks of relapse [5,6]. Some institutions demonstrated HID-SCT using conditioning regimen including ATG yielded similar outcomes to ISD-SCT for hematological malignancies [7,8]. Some studies also indicated similar outcomes after HID-SCT compared to HLA-identical allo-HSCT [9,10]. At present, HID-SCT has been accepted by many transplantation centers. Over the past decades, much progress has been made to improve the outcomes of transplantation, including in the conditioning regimen; prophylaxis; lower cumulative incidence rates of GVHD, transplantation-related mortality/ no-relapse mortality (TRM/NRM) and relapse; higher rates of OS and DFS/LFS/PFS. However, there haven’t been well-controlled studies to compare the efficacy of HID-SCT and ISD-SCT. Therefore, our meta-analysis aims to investigate whether HID-SCT has similar outcomes compared with ISD-SCT.

## Methods

### Identification and study selection

Two reviews independently identified relevant studies by searching PubMed. Search terms included “haploidentical stem cell transplantation”, “haploidentical” and “identical”. All studies published prior to February 2017 were eligible. The title and abstracts of all potentially relevant publications were reviewed. Studies that met the inclusion criteria were selected for the analysis. The reference lists from the selected articles were then hand-searched to identify further relevant trials.

### Inclusion and exclusion criteria

This meta-analysis included hematologic malignancies who received HSCT (HID-SCT or ISD-SCT). T cell replete HID-SCT for hematologic malignancies using GVHD prophylaxis (CsA + MMF + MTX + ATG) was included. Studies with data concerning grades 3–4 acute GVHD were included if no suitable data of grades 2–4 acute GVHD were available. The inclusion criteria were as follows: randomized/nonrandomized studies that compared the outcomes of HID-SCT versus ISD-SCT; the key outcomes including engraftment, OS, DFS/LFS/RFS, acute/chronic-GVHD, relapse and NRM/TRM; myeloablative conditioning regimens and HID group GVHD prophylaxis (CsA + MMF + MTX + ATG).

We excluded ongoing studies or studies with data inaccessible. If the same authors had more than one publication based on same population, only the most recent or most complete report was included.

### Quality assessment

Two reviewers independently assessed the quality of the included studies using Newcastle-Ottawa scale [11]. Studies scoring more than 5 stars were considered acceptable. Data were independently abstracted by each reviewer. Any disagreement between the two reviewers was solved by a third person who extracted the data again.

### Statistical analysis

The extracted information was analyzed on the Cochrane statistical program Review Manager 5.3. For the key outcomes, odds ratio (OR) and 95% confidence intervals (CI) were calculated for each trial. Dichotomous outcomes were determined by the number of participants with events and the total number of participants in HID-SCT and ISD-SCT. Heterogeneity was checked by Q-test and defined as P < 0.1. Heterogeneity was quantified using the *I*^2^ metric (*I*^2^<50% acceptable level of heterogeneity; *I*^2^>50%, large or extreme). We performed meta-analysis using a fixed or random effect model (Mantel–Haenszel method for dichotomous data). A funnel plot was applied to detect the presence of publication bias.

## Result

### Description of included studies

**Fig 1:**
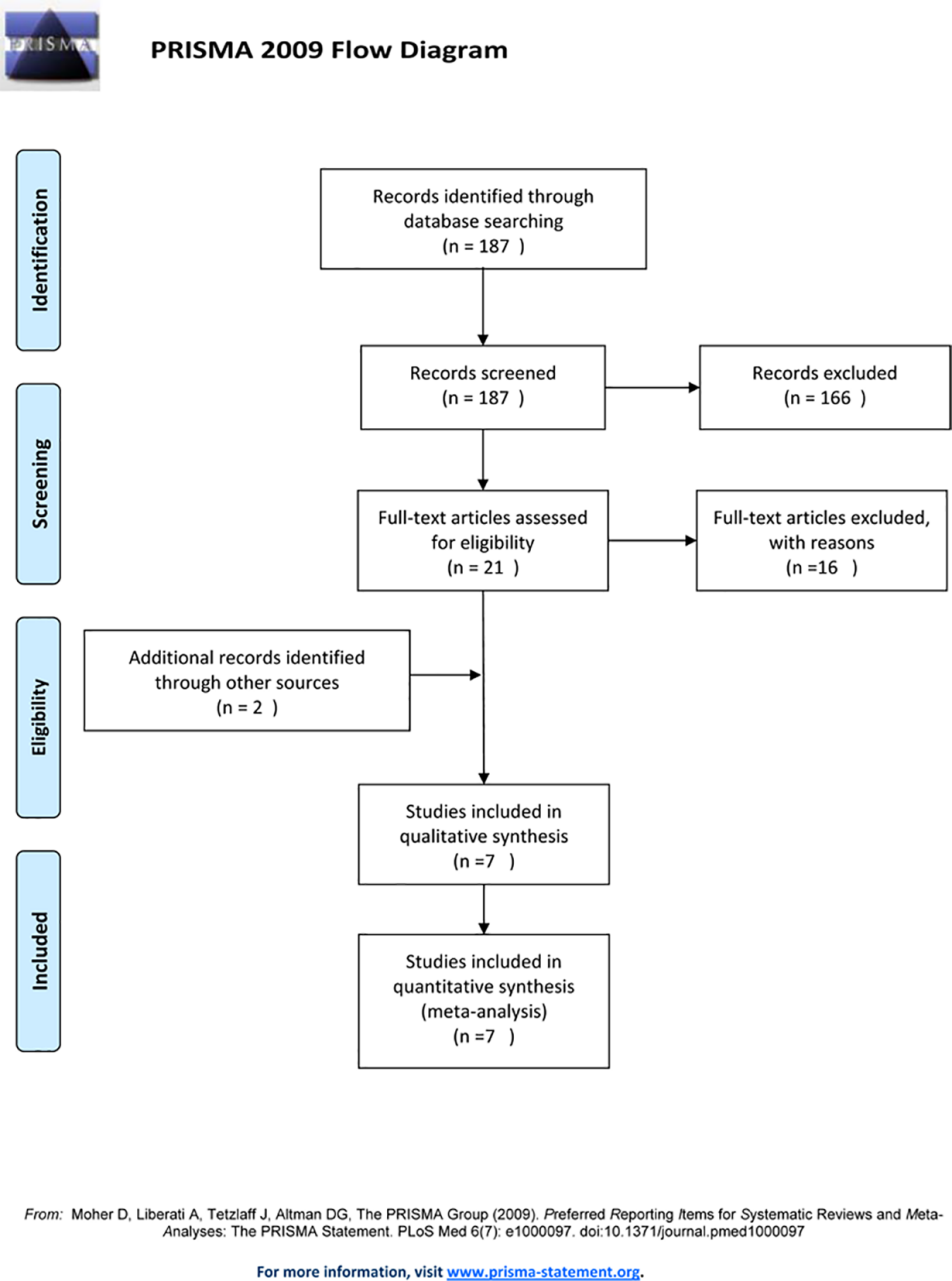
Flowchart of selection of studies for inclusion in meta-analysis.

A total of 187 potentially relevant publications were retrieved from our initial search (**Fig 1**). Among these, 46 publications were excluded for review, 120 studies were excluded for not fulfilling the inclusion criteria and 16 were excluded for meeting the exclusion criteria. Additionally, 2 articles were included through searching the reference lists. Finally, 7 studies including 1919 patients were included [7,8,12–16] (**Table 1**). Specifically, 936 patients were treated with HID-SCT and 983 patients received ISD-SCT. Both non-randomized and non-blinded comparative studies were included. All data from the comparative studies of HID-SCT versus ISD-SCT were clinical trials. The key outcomes were neutrophil and platelet engraftment, OS, DFS/LFS/RFS, acute/chronic-GVHD, relapse and NRM/TRM, myeloablative conditioning regimen and HID group with GVHD prophylaxis (CsA + MMF + MTX + ATG) were also included.

**Table 1:** Study quality.

Study	Selection	Comparability	Outcome
QF Liu(2015)	**	**	**
Xh Chen(2009)	**	**	**
Yi Luo(2014)	**	**	**
Y Wang(2016)	**	**	**
SuJian Yu(2016)	**	**	**
YU Wang(2016)	**	**	**
Daopei Lu(2006)	**	**	**

A study can be awarded a maximum of one star for each numbered item within the Selection and Outcome categories if it meets the criteria. A maximum of two stars can be given for Comparability.

Selection: 1) Representativeness of the exposed cohort: truly/somewhat representative of the average in the community; 2) Selection of the non exposed cohort: drawn from the same community as the exposed cohort; 3) Ascertainment of exposure: secure record (eg surgical records) or structured interview; 4) Demonstration that outcome of interest was not present at start of study.

Comparability: 1) Comparability of cohorts on the basis of the design or analysis: a) stdy controls for; b) study controls for additional factor.

Outcome: 1) Assessment of outcome: independent blind assessment or record linkage; 2) The follow-up was long enough for outcomes to occur; 3) Adequacy of follow up of cohorts: complete follow up–all subjects accounted for, or subjects lost to follow up unlikely to introduce bias.

### Characteristics of the included studies and subgroups

The characteristics of all included studies are shown in **Table 2**. Different hematologic diseases were included, such as acute myelogenous leukiemia (AML), acute lymphocytic leukemia (ALL), myelodysplastic syndromes (MDS), chronic myelogenous leukemia (CML), and lymphoma. Subgroups were divided by the follow up time ≤ 3 year and ≥ 4 year. The outcomes of each study are shown in **Table 3**.

**Table 2:** Characteristics of included studies.

Trials	Type	Number	age	Diagnosis	Risk Status	Conditioning regimen		GVHD prophylaxis
QF Liu(2015)[12]	HID	231	28(15–57)	AML(231)	CR(231)	BUCY(231)	MAC	CsA+MMF+MTX+ATG
	ISD	219	40(17–60)	AML(231)	CR(219)	BUCY(219)	MAC	CsA+MMF+MTX
Xh Chen(2009)[13]	HID	46	25(5–54)	AML(15);ALL(14);CML(17)	CR+CP(39);Ad(7)	TBI-based(34);BUCY(12)	MAC	CsA+MMF+MTX+ATG
	ISD	52	30(15–52)	AML(12);ALL(11);CML(29)	CR+CP(44);Ad(8)	TBI-based(21);BUCY(31)	MAC	CsA+MMF+MTX
Yi Luo(2014)[7]	HID	99	25(9–55)	AML(42);ALL(32);CML(5);MDS(7); NHL(4)	CR(78);Ad(6)	BUCY(99)	MAC	CsA+MMF+MTX+ATG
	ISD	90	33.5(16–56)	AML(29);ALL(50);CML(5);MAPL(2); MDS(10);NHL(3)	CR(71);Ad(7)	BUCY(90)	MAC	CsA+MMF+MTX
Y Wang(2016)[14]	HID	226	30-35(4–61)	MDS(226)	CR(21);Ad(49)	BUCY(226)	MAC	CsA+MMF+MTX+ATG
	ISD	228	40(4–61)	MDS(228)	CR(40);Ad(51)	BUCY(228)	MAC	CsA+MMF+MTX
SuJian Yu(2016)[15]	HID	96	25(12–54)	AML(40);ALL(40);ABL(10);CML(6)	CR(58);Ad(38)	BUCY(35);BUFlu(2); TBI-based(59)	MAC	CsA+MMF+MTX+ATG
	ISD	153	31(12–61)	AML(72);ALL(62);ABL(13);CML(6)	CR(122);Ad(31)	BUCY(34);BUFlu(25); TBI-based(94)	MAC	CsA/CsA+MTX
YU Wang(2016)[16]	HID	121	26(18–59)	ALL(121)	CR(121)	BUCY(121)	MAC	CsA+MMF+MTX+ATG
	ISD	89	38(18–59)	ALL(121)	CR(89)	TBI-based(21);BUCY(62)	MAC	CsA+MMF+MTX
Daopei Liu(2006)[8]	HID	135	24 (3–50)	CML(43);AML(30);ALL (53);MDS(10)	CR1+CP(68);Ad(67)	BUCY(135)	MAC	CsA+MMF+MTX+ATG
	ISD	158	37 (5–50)	CML(68);AML(39);ALL (39);MDS(12)	CR1+CP(100);Ad(58)	BUCY(158)	MAC	CsA+MMF+MTX CsA+MMF+MTX+ATG(7)

HID = haploidentical donor, ISD = HLA-identical sibling donor, CML = chronic myeloid leukemia, AML = acute myeloid leukemia, ALL = acute lymphoid leukemia, MDS = myelodysplastic syndrome, MPAL = acute mixed phenotypic leukemia, NHL = non-Hodgkin's lymphoma, ABL = acute biphenotypic leukemia, CR = complete remission, CP = chronic phase, Ad = advance disease(no-CR,no-CP), BU = busulfan, CY = cyclophosphamide, TBI = total body irradiation,MAC = myeloablative conditioning, CsA = cyclosporine A, MMF = mycophenolate mofetil, MTX = methotrexate, ATG = antithymoglobulin, Flu = fludarabine.

**Table 3:** Outcome of included studies.

Trails	type	Patients (n)	Neutrophil Engraftment(n)	Platelet engraftment(n)	acute GVHD(n)	Chronic GVHD (n)	Relapse (n)	NRM(n)	TRM(n)	DFS/LFS/RFS (n)	OS (n)
QF Liu(2015)[12]	HID	231	30d(231)	100d(208)	Grades 3–4 100d(24)	1y(97)	3y(35)	3y(30)	-	DFS 3y(171)	3y(183)
	ISD	219	30d(219)	100d(211)	Grades 3–4 100d(7)	1y(33)	3y(33)	3y(18)	-	DFS 3y(171)	3y(180)
Xh Chen(2009)[13]	HID	46	30d(46)	30d(46)	Grades 3–4 100d(0)	(5)	2y(11)	-	2y(4)	LFS 2y(33)	2y(36)
	ISD	52	30d(52)	30d(52)	Grades 3–4 100d(0)	(18)	2y(10)	-	2y(5)	LFS 2y(38)	2y(40)
Yi Luo(2014)[7]	HID	99	30d(99)	30d(95)	Grades 3–4 3m(17)	2y(41)	5y(14)	5y(31)	-	DFS 5y(58)	5y(61)
	ISD	90	30d(90)	30d(86)	Grades 3–4 3m(5)	2y(22)	5y(31)	5y(5)	-	DFS 5y(58)	5y(70)
Y Wang(2016)[14]	HID	226	28d(216)	100d(182)	Grades 3–4 100d(17)	4y(91)	4y(15)	4y(73)	-	RFS 4y(136)	4y(136)
	ISD	228	28d(216)	100d(208)	Grades 3–4 100d(16)	4y(117)	4y(23)	4y(37)	-	RFS 4y(162)	4y(167)
Sijian Yu(2016)[15]	HID	96	-	-	Grades 3–4 100d(39)	2y(51)	5y(19)	-	5y(26)	DFS 5y(58)	5y(58)
	ISD	153	-	-	Grades 3–4 100d(36)	2y(66)	5y(41)	-	5y(27)	DFS 5y(90)	5y(99)
YU Wang(2016)[16]	HID	103	30d(102)	100d(91)	Grades 3–4 100d(7)	3y(40)	3y(19)	3y(14)	-	DFS 3y(70)	3y(78)
	ISD	83	30d(82)	100d(81)	Grades 3–4 100d(2)	3y(21)	3Y(20)	3y(10)	-	DFS 3y(54)	3y(59)
Daopei Liu(2006)[8]	HID	135	-	-	Grades 2–4 100d(54)	2y(66)	2y(18)	-	2y(20)	LFS 2y(87)	2y(96)
	ISD	158	-	-	Grades 2–4 100d(51)	2y(83)	2y(29)	-	2y(23)	LFS 2y(113)	2y(114)

d = day, m = month, y = year, NRM = no relapse mortality, DFS = diseae free survival, LFS = leukemia free survival, RFS = relapse free survival, TRM = treatment-related mortality, OS = overall survival, GVHD = graft versus host disease

## Effects of interventions

### Neutrophil and platelet engraftment

**Fig 2:**
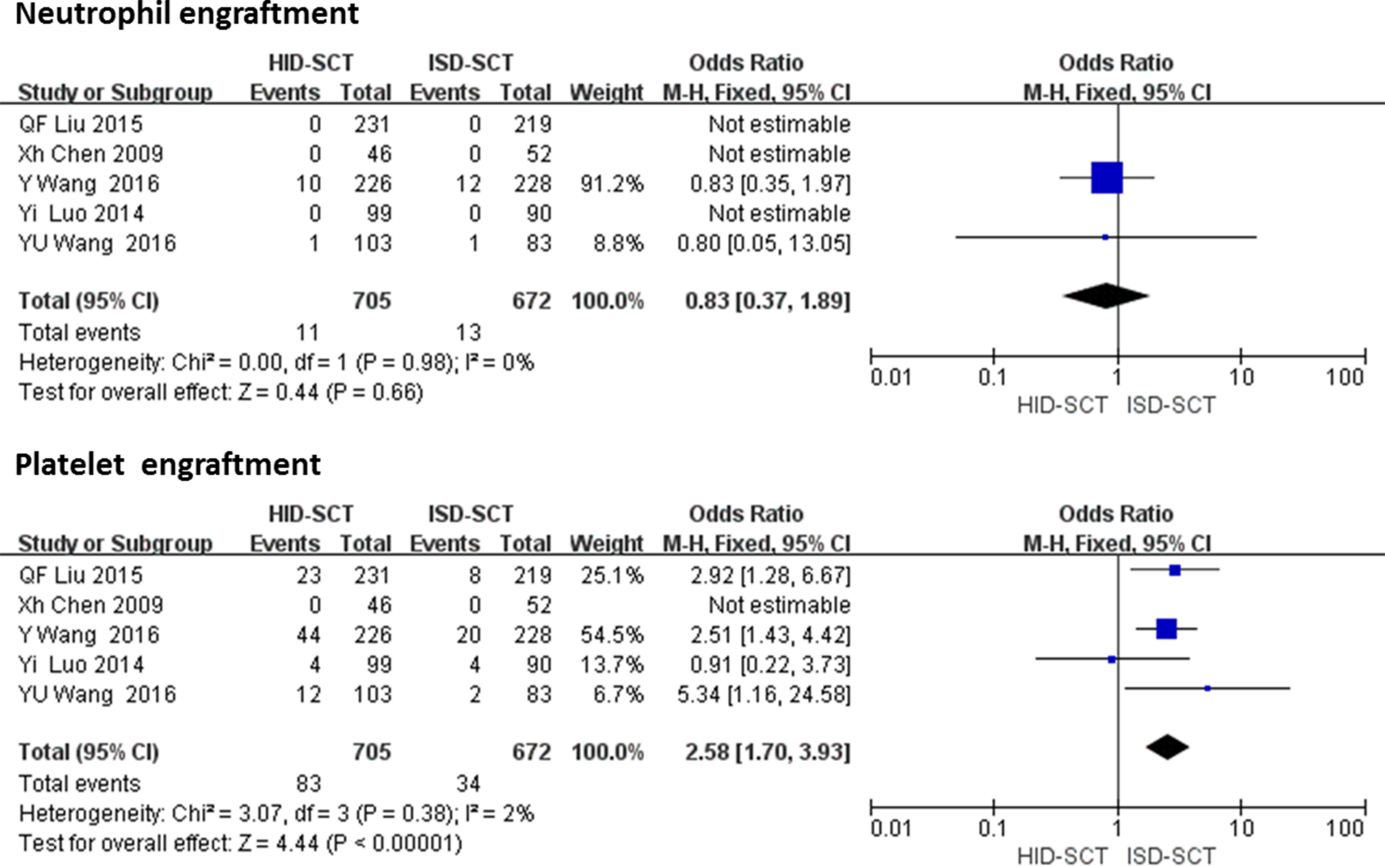
Forest plot of comparisons between HID-SCT and ISD-SCT: Neutrophil and platelet engraftment.

Five studies reported neutrophil and platelet engraftment rates, including 705 HID-SCT-treated patients and 672 ISD-SCT-treated patients. For neutrophil engraftment, a fixed effect model was used. There were no significances differences in the incidence rates of neutrophil engraftment between the two groups (OR = 0.83, 95% CI = 0.37–1.89, *P* = 0.66, *I*^2^ = 0). The platelet engraftment was significantly faster following ISD-SCT than HID-SCT (OR = 2.58, 95% CI = 1.70–3.93, *P <* 0.00001, *I* ^2^ = 30%) (**Fig 2**).

### GVHD

**Fig 3:**
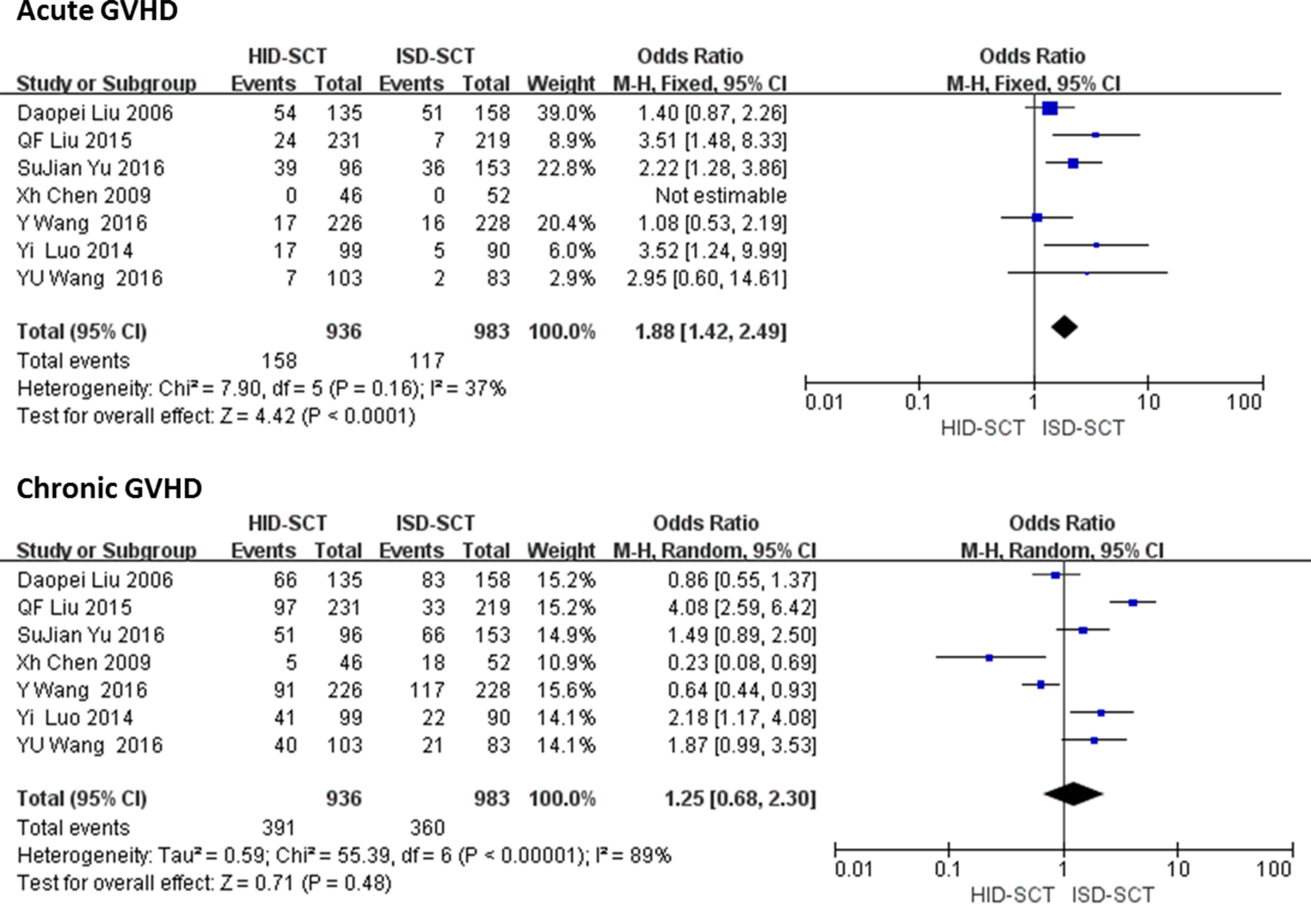
Forest plot of comparisons between HID-SCT and ISD-SCT: Acute GVHD and chronic GVHD.

There is a significant difference between HID-SCT and ISD-SCT regarding the incidence rates of acute GVHD, but not chronic GVHD. We extracted data regarding acute GVHD from seven studies including 1919 patients. The fixed effect model was used, results showed the risk of acute GVHD after HID-SCT was significantly higher than ISD-SCT (OR = 1.88, 95% CI = 1.42–2.49, *P* < 0.00001, *I*^2^ = 37%). No significant difference was found in the incidence rates of chronic GVHD between HID-SCT and ISD-SCT (OR = 1.25, 95% CI = 0.68–2.30, *P* = 0.48, *I*^2^ = 89%) (**Fig 3**).

### Relapse and NRM/TRM

**Fig 4:**
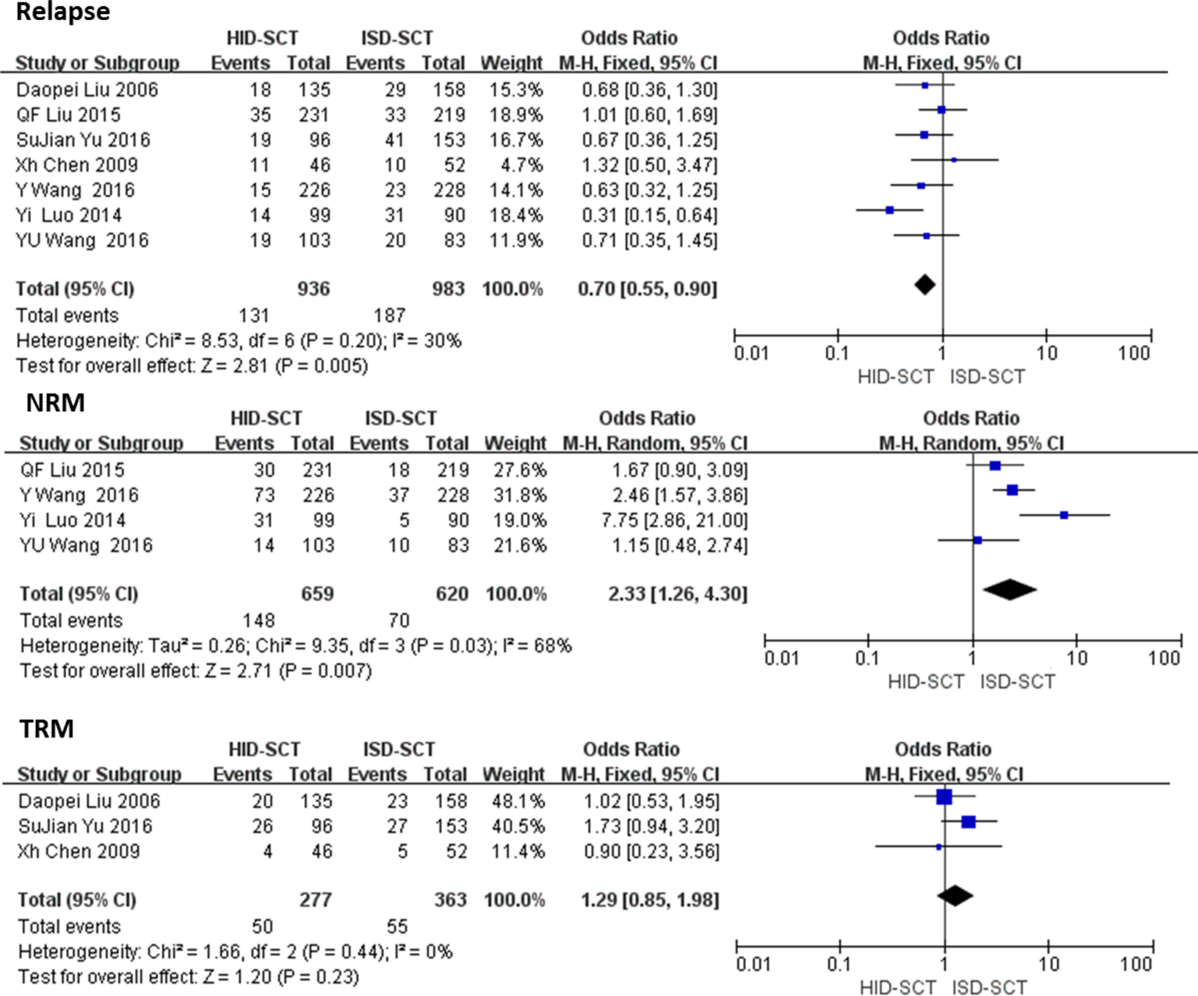
Forest plot of comparisons between HID-SCT and ISD-SCT: Relapse and NRM/TRM.

Seven studies regarding relapse involving 1919 patients were analyzed with the fixed effect model. The risk of relapse after HID-SCT was significantly lower than ISD-SCT (OR = 0.70, 95% CI = 0.55–0.90, *P* = 0.005, *I*^2^ = 30%). Four studies reported the NRM rates and three reported TRM rates, respectively. The risk of TRM was not significantly different between HID-SCT-treated and ISD-SCT-treated patients (OR = 1.29, 95% CI = 0.85–1.98, *P* = 0.23, *I*^2^ = 0). Significant difference in TRM was found, but it was unreliable because of unacceptable heterogeneity (OR = 2.33, 95% CI = 2.16–4.30, *P* = 0.007, *I*^2^ = 68%) (**Fig 4**).

### DFS/LFS/RFS and OS

**Fig 5:**
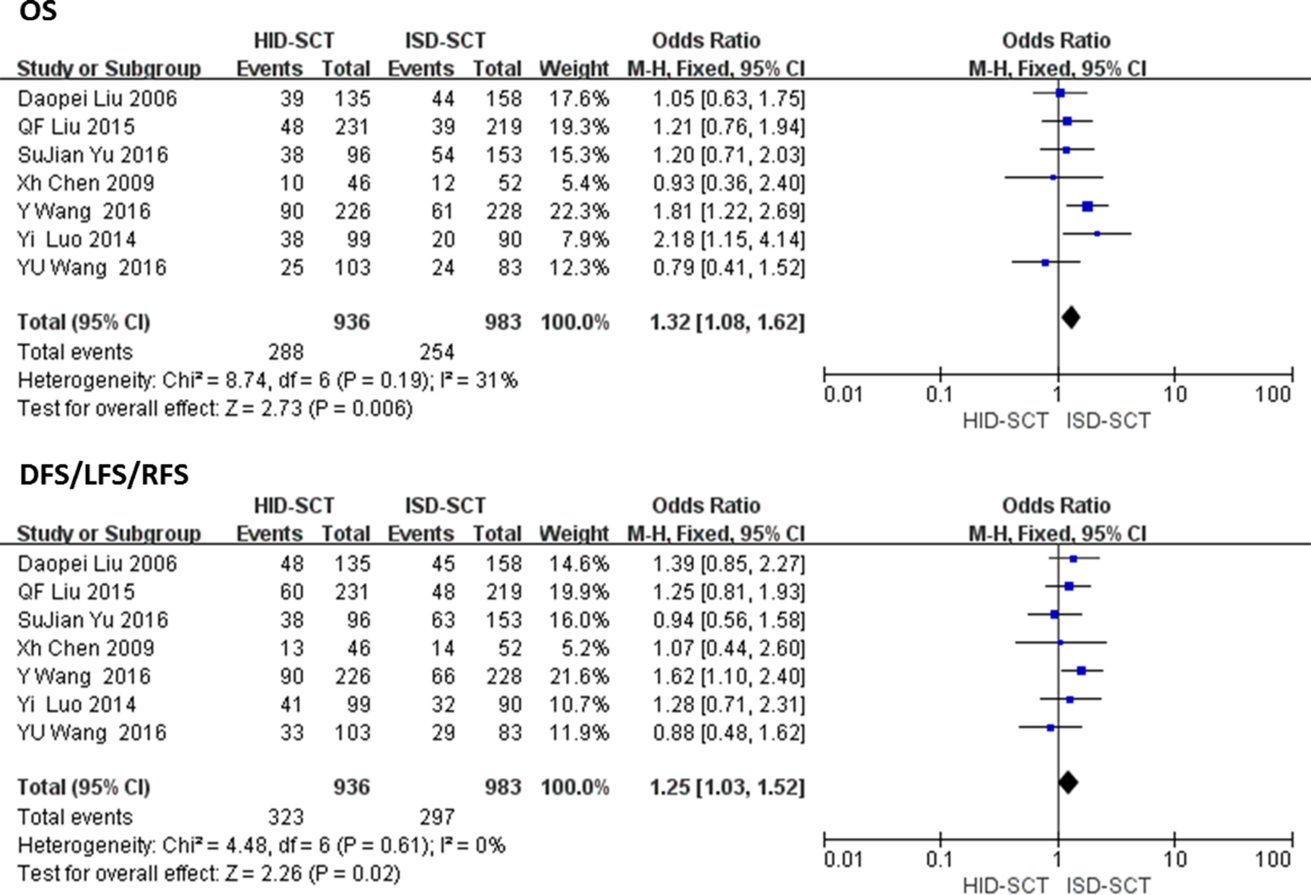
Forest plot of comparisons between HID-SCT and ISD-SCT: DFS/LFS/RFS and OS.

All seven studies reported DFS/LFS/RFS and OS. Fixed effect model showed the heterogeneity of outcomes was acceptable. The rates of DFS/LFS/RFS after HID-SCT were significantly lower compared with ISD-SCT (OR = 1.25, 95% CI = 1.03–1.52, *P* = 0.02, *I*^2^ = 0). Significant differences in OS and longer life expectancy were found between ISD-SCT-treated and HID-SCT-treated group (OR = 1.32, 95% CI = 1.08–1.62, *P* = 0.006, *I*^2^ = 31%) (**Fig 5**).

## Meta regression and subgroup analysis

**Fig 6:**
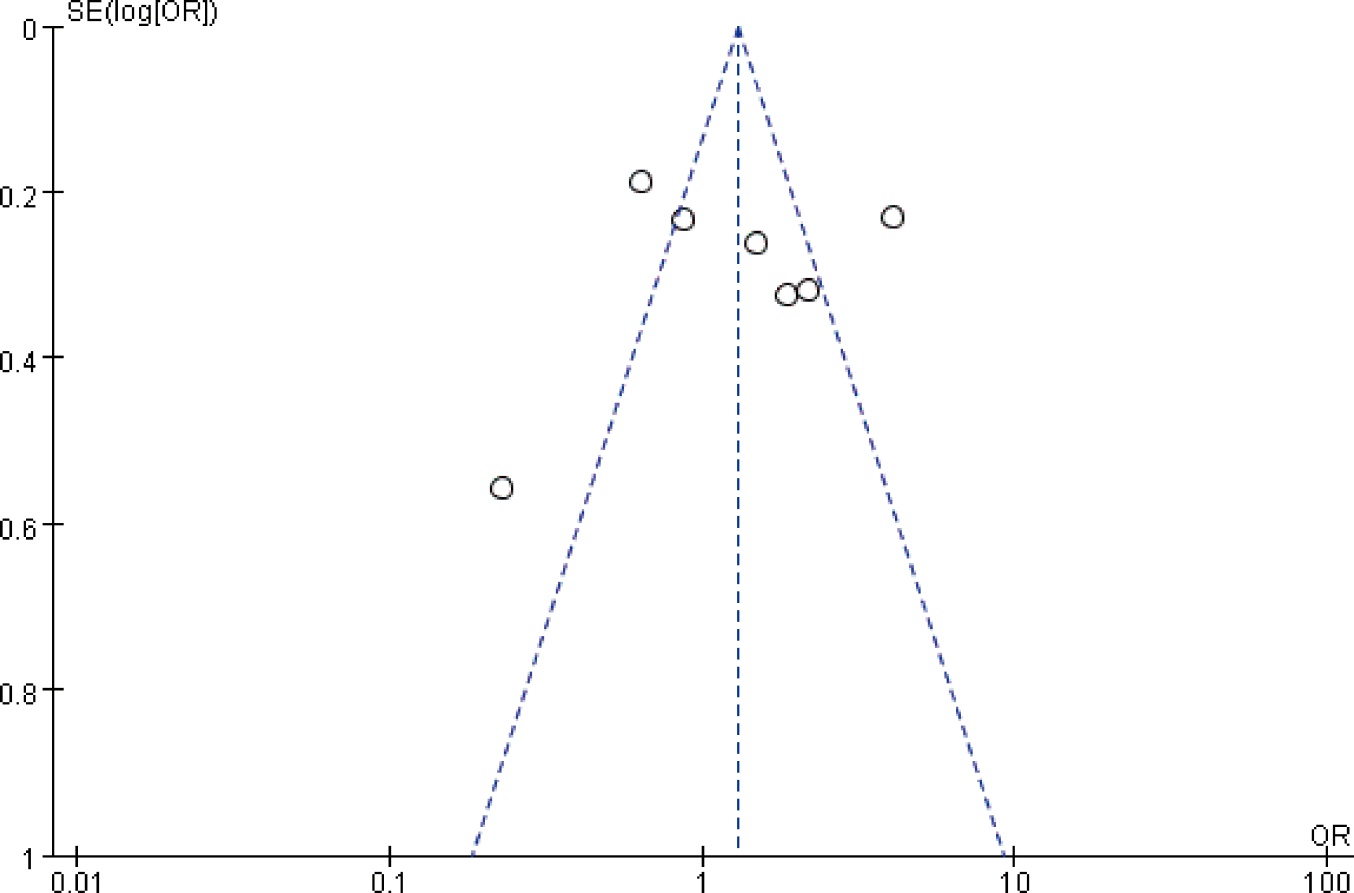
Funnel plot of comparisons between HID-SCT and ISD-SCT: Chronic GVHD.

As mentioned above, we found significant heterogeneity in chronic GVHD and NRM rates. Thus, subgroup analysis by follow-up time was performed to further investigate NRM. It was found the cumulative incidence rates of NRM for HID-SCT and ISD-SCT were similar at ≤ 3 year, but not at ≥ 4year (*P =* 0.13, *I*^2^ = 0; *P =* 0.01, *I* ^2^ = 77%) (**Table 4**). For chronic GVHD, publication bias might cause the heterogenity(**Fig 6**).

**Table 4:** P value and 95% CI of subgroup for NRM.

outcome	subgroup	studies	patients	OR	95% CI	P	*I*^2^
NRM							
Follow-up time	≤3 years	2	636	1.47	0.89–2.43	0.13	0
	≥4 years	3	643	4.00	1.31–12.2	0.01	77

NRM = transplantation related mortality, OR = odds ratio, CI = confidence intervals

## Discussion

To our knowledge, this is the first meta-analysis that compares HID-SCT with ISD-SCT in an unselected population of hematological malignancies. The results indicate that HID-SCT is associated with a higher risk for acute GVHD, a lower rate of platelets engraftment, and worse OS and DFS/LFS/RFS. No significant difference was found in TRM rate and neutrophil engraftment. Previous studies reported that HID-SCThad lower neutrophil engraftment rate which led to higher TRM. Also the median time to engraftment after HID-SCT was significantly longer than after ISD-SCT [7,8,9,12,15]. HID-SCT without ATG resulted in similar median time for neutrophil engraftment, and longer platelet engraftment compared with ISD-SCT [17]. ATG is associated with delayed immune reconstitution, which is often accompanied by severe infections and higher TRM. But we found no significant difference in neutrophil engraftment (according to the follow-up time (30 days)) or cumulative incidence of TRM between the two groups. Although the median time to engraftment in HID-SCT group was significantly longer than in the ISD-SCT group, it may indicate neutrophil engraftment within 30d, did not increase the risk of TRM. For platelet engraftment, whatever median time or 100d cumulative incidence, HID-SCT was thought to have lower engraftment rate than ISD-SCT [7,12,14–16]. Our meta-analysis indicates HID-SCT is associated with a higher risk of acute GVHD. However, the reduced intensity transplantation (RIC) with HID-SCT (post-transplantation cyclophosphamide PT-Cy) has showed similar risks of severe acute GVHD and 3-year rates of NRM, relapse, OS and PFS compared with ISD-SCT [18]. Similarly, HID-SCT (T-cell depleted grafts or CsA + MMF + Cyclophosphamide) was associated with a similar risk of severe acute GVHD [17,19]. However, some studies reported a higher risk of grade 3–4 of acute GVHD following HID-SCT with ATG compared to ISD-SCT [7,12,15] while some other studies found a similar risk of grade 3–4 of GVHD between HID-SCT with ATG and ISD-SCT [13,14]. Our meta-analysis indicates HID-SCT with ATG has a higher risk of serious acute GVHD than ISD-SCT. Further studies are needed to uncover whether ATG can reduce the risk of severe acute GVHD compared with T-cell depleted grafts or PT-Cy. Higher risk for GVHD is associated with more mortality, correspondingly, the low rate of relapse is supposed to improve DFS/LFS/RFS, even OS. It should be mentioned that relevant studies also suggested in high risk acute leukemia, HID-SCT has lower relapse rate and longer OS than ISD-SCT [20]. Many studies showed the cumulative risk of relapse was similar or lower after HID-ISD than ISD-SCT [7,8,12,14–16]. However, this finding is not fully consistent with the conclusions of our meta-analysis. We found a lower risk for relapse after HID-SCT, but did not find higher rates for DFS/LFS/RFS or OS. HID-SCT may be superior to ISD-SCT in terms of high risk acute leukemia but no evidence suggests the low risk of relapse could lead to better DFS/LFS/RFS or OS.

For the incidence rates of NRM after HID-SCT and ISD-SCT, the outcomes of NRM were opposite between the follow up durations ≤ 3 years and ≥ 4 years. We did not find significant differences between the two groups at the follow up time ≤ 3 years. Related research also showed no significant differences in the incidence of NRM at one year [20]. However, the 2-year incidence of NRM was significantly lower among haploidentical related recipients compared to HLA-matched related recipients for relapsed or refractory Hodgkin Lymphoma [21]. Over the past decades, new approaches (such as GVHD prophylaxis, conditioning regimen) were applied to HID-SCT which have effectively controlled intense alloreactivity, resulting in improved outcomes, but our results indicate ISD-SCT is still the preferred option in all transplantations, and other promising HID-SCT strategies should be pursued.

Our meta-analysis has some limitations, such as different risk status and diagnosis among the included studies, long time interval, and different follow up durations. To obtain better conclusion, we need more randomized controlled studies.

## Supporting information

S1 Table PRISMA checklist 2009. (DOC)
