# Supplementary material for: Comparison of outcomes in hematological malignancies treated with haploidentical or HLA-identical sibling hematopoietic stem cell transplantation following myeloablative conditioning: A meta-analysis
Source: PLoS One. 2018 Jan 30;13(1):e0191955. doi: 10.1371/journal.pone.0191955 (PMC5790250; doi:10.1371/journal.pone.0191955)
Supplement: S1 Table — (DOC) [file pone.0191955.s001.doc]

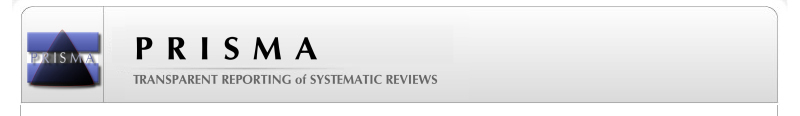
**PRISMA 2009 Flow Diagram**

**Screening**

**Included**

**Eligibility**

**Identification**

Records identified through database searching
(n = 187 )

Additional records identified through other sources
(n = 2 )

Records screened
(n = 187 )

Records excluded
(n = 166 )

Full-text articles assessed for eligibility
(n = 21 )

Full-text articles excluded, with reasons
(n =16 )

Studies included in qualitative synthesis
(n =7 )

Studies included in quantitative synthesis (meta-analysis)
(n =7 )
